# The Origin of the Intracellular Silver in Bacteria: A Comprehensive Study using Targeting Gold–Silver Alloy Nanoparticles

**DOI:** 10.1002/adhm.202302084

**Published:** 2023-09-17

**Authors:** Carmen Streich, Frederic Stein, Jurij Jakobi, Alexandra Ingendoh‐Tsakmakidis, Nils Heine, Christoph Rehbock, Andreas Winkel, Sebastian Grade, Mark Kühnel, Vadim Migunov, András Kovács, Thomas Knura, Meike Stiesch, Bernd Sures, Stephan Barcikowski

**Affiliations:** ^1^ University Duisburg‐Essen Technical Chemistry I, Universitaetsstr. 7 45141 Essen Germany; ^2^ Department of Prosthetic Dentistry and Biomedical Materials Science Hannover Medical School Carl‐Neuberg‐Straße 1 30625 Hannover Germany; ^3^ Ernst Ruska‐Centre for Microscopy and Spectroscopy with Electrons Forschungszentrum Jülich 52425 Jülich Germany; ^4^ University Duisburg‐Essen Aquatic Ecology Universitaetsstr. 5 45141 Essen Germany

**Keywords:** AgAu, alloy particles, aptamers, biomedicine, LAL, nano‐bioconjugates

## Abstract

The bactericidal effects of silver nanoparticles (Ag NPs) against infectious strains of multiresistant bacteria is a well‐studied phenomenon, highly relevant for many researchers and clinicians battling bacterial infections. However, little is known about the uptake of the Ag NPs into the bacteria, the related uptake mechanisms, and how they are connected to antimicrobial activity. Even less information is available on AgAu alloy NPs uptake. In this work, the interactions between colloidal silver–gold alloy nanoparticles (AgAu NPs) and *Staphylococcus aureus* (*S. aureus*) using advanced electron microscopy methods are studied. The localization of the nanoparticles is monitored on the membrane and inside the bacterial cells and the elemental compositions of intra‐ and extracellular nanoparticle species. The findings reveal the formation of pure silver nanoparticles with diameters smaller than 10 nm inside the bacteria, even though those particles are not present in the original colloid. This finding is explained by a local RElease PEnetration Reduction (REPER) mechanism of silver cations emitted from the AgAu nanoparticles, emphasized by the localization of the AgAu nanoparticles on the bacterial membrane by aptamer targeting ligands. These findings can deepen the understanding of the antimicrobial effect of nanosilver, where the microbes are defusing the attacking silver ions via their reduction, and aid in the development of suitable therapeutic approaches.

## Graphical abstract

This study uses targeting silver‐gold alloy nanoparticles to show that silver nanoparticles found inside bacteria follow a RElease‐PEnetration‐Reduction (REPER) mechanism of the emitted silver cations, with the microbes thereby potentially defusing the therapeutic effect related to silver ions.

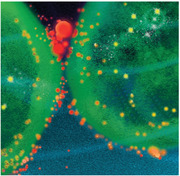

## 1 Introduction

Colloidal nanoparticles (NPs) with alloyed silver and gold (AgAu) can combine the attractive chemical, biological, and plasmonic characteristics of both materials and even result in new properties.^[^ ^1^ ^]^ In these hybrid particles, gold is interesting due to its chemical inertness resulting in high biocompatibility. Furthermore, gold surfaces can be easily conjugated to functional biomolecules via thiol‐gold chemistry.^[^ ^2^ ^]^ Silver, on the other hand, as a second constituent of an AgAu NP, features an even higher molar extinction coefficient than gold at the surface plasmon resonance (SPR) peak wavelength and shows antibacterial properties due to the dissolution and release of Ag^+^ cations.^[^ ^3^ ^]^ The formation of alloy NPs thus allows the combination of both elements’ beneficial traits; however, their biological response needs to be evaluated. In this regard, previous studies with alloy NPs of different AgAu ratios detected cytotoxic and antimicrobial effects disproportional to the silver content.^[^ ^4^ ^]^ Surprisingly high toxicity was found for Ag_80_Au_20_ alloys, which was even more substantial than the effect of pure Ag NPs.^[^ ^5^, ^6^ ^]^ On the other hand, for alloy NPs with gold fractions <50%, antibacterial and cytotoxic effects decreased unexpectedly.^[^ ^5^, ^6^, ^7^ ^]^ For Ag_50_Au_50_ alloy NPs, human mesenchymal stem cell activation was observed, but alloy NPs did not impair cell viability in contrast with pure Ag NPs.^[^ ^8^ ^]^ A possible mechanistic explanation for the alleviation of silver cytotoxicity is that alloying with gold may drastically minimize the surface oxidation and leaching of toxic Ag^+^ cations,^[^ ^9^, ^10^ ^]^ which may result from alterations in the entropy of mixing, or composition changes in the course of Ag^+^ release, altering the alloy particle's redox potential.^[^ ^11^, ^12^, ^13^ ^]^

While the bactericidal effects of AgAu alloy nanoparticles are relatively well examined, studies on their cellular uptake by bacteria are unexplored. Thereto, we will briefly review what is known about the bacterial uptake of metal NPs in general. Morones et al. studied the effect of Ag NPs on gram‐negative bacteria showing that particles with *d* < 10 nm not only attached to the membrane but also penetrated the bacteria and distributed throughout the cell.^[^ ^14^ ^]^ Salopek et al. suggested that Ag NPs may interact with the membrane, causing structural changes (“pits”) and degradation of gram‐negative *Escherichia coli*.^[^ ^15^ ^]^ It was further suggested that different membrane structures of gram‐positive and gram‐negative bacteria (i.e., peptidoglycan thickness) could cause deviating antimicrobial and uptake properties.^[^ ^16^ ^]^ However, to date, it remains unclear whether or to what extent nanoparticle uptake is involved in antimicrobial activity. Ag^+^ cations rather than solid Ag are commonly believed to be the toxic species, and internalization may not be required for toxicity per se.^[^ ^17^ ^]^ Sawosz et al. studied the uptake of Au and Pt NPs by *Salmonella enteritidis* (gram‐negative) and *Listeria monocytogenes* (gram‐positive).^[^ ^18^ ^]^ A material‐dependent internalization was observed, in which Pt NPs but not Au NPs could penetrate the cell wall and cytoplasmic membrane of both microorganisms.

Considering the previously described results, two general conclusions may be drawn. The gram‐positive bacterial wall consisting of thicker peptidoglycan layers compared with gram‐negative strains seems to impede NP internalization.^[^ ^19^ ^]^ Moreover, it remains to be clarified if NP uptake is generally difficult for bacteria.^[^ ^20^ ^]^

In the present study, AgAu alloy NPs were incubated with bacterial *S. aureus* cells, and Au NPs were used as controls. To investigate the influence of material composition on cellular uptake mechanisms, Au NPs and AgAu alloy NPs were generated by pulsed laser ablation in liquid (PLAL) from bulk Au or AgAu 50/50 bulk alloy foils.^[^ ^21^ ^]^ Laser ablation allows the fabrication of colloidal NPs without conventional artificial ligands, reducing agents, or metal precursor salts, thus creating highly pure particles and avoiding cross effects during biological applications that may otherwise arise from residual additives of the chemical synthesis.^[^ ^22^ ^]^ Subsequently, the particles were functionalized with aptamers, specifically targeting the bacterium *S. aureus*.^[^ ^23^ ^]^ Here, we deliberately chose Ag_50_Au_50_ NPs with a relatively high gold content, which only shows low overall antimicrobial activity,^[^ ^7^ ^]^ to rule out potential alternate uptake mechanisms derived from membrane disintegration in dead bacteria. For the same reason, we also excluded similarly fabricated pure AgNPs from the biological studies as their innate cytotoxic effects damage the bacterial cell membrane, which makes systematic uptake studies impractical. A set of control experiments, including the incubation withAg ions (in prersence and absence of the targeting ligand) as well as ion reduction and ion release analyses, complement the present study.

## 2 Results and Discussion

### 2.1 Nanoparticle Analysis

**Figure 1:**
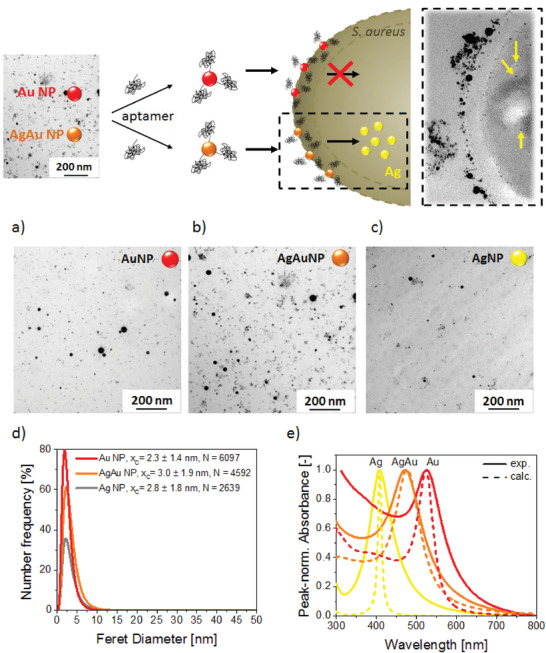
Study design (top) and characterization of laser‐generated nanoparticles (bottom). Pure metal and alloy NPs are generated from metal and alloy foils by pulsed laser ablation in an aqueous solution. Transmission electron micrographs of: a–c) as‐synthesized particles, primary particle size distributions obtained from TEM analysis (Curves represent the number‐weighted size distributions fitted with a log‐normal function using N individual particles as a basis. The *x*_c_ represents the number‐mean values of the distributions and errors are derived from the variance of the log‐normal fit function) (d), and exemplary UV/Vis extinction spectra measured by UV–Vis spectroscopy and calculated using Mie‐Plot software (e).

Colloidal nanoparticle‐aptamer conjugates were characterized according to their size and optical properties (**Figure** **1**). Transmission electron microscopy (TEM) micrographs revealed average particle diameters of 2–3 nm for both Au NPs and AgAu alloy NPs (Figure 1d). In all cases, number‐weighted particle size distributions were created from >2500 particles per sample, and mean particle diameters (*x*_c_) and variance were calculated from a log‐normal fit of the corresponding size distributions. The small size of the laser‐generated particles (*d* < 10 nm) results from a size quenching process through albumin molecules present in the aqueous ablation medium, which sterically impede particle growth during laser ablation similar to the size quenching by oligonucleotides or small peptides.^[^ ^24^ ^]^ The optical properties of the AgAu alloy NP conjugates were analyzed via ultraviolet–visible (UV/Vis) extinction spectroscopy (Figure 1e), while theoretical values were calculated using Mie‐Plot.^[^ ^25^ ^]^ The distinct, single peak at 471 nm indicates the formation of alloy NPs with a characteristic SPR peak in between the peaks of the pure metals. Peaks were found neither at 407 nm nor at 523 nm, indicating that no significant amounts of single metal Ag NPs or Au NPs were formed (compare reference spectra of the pure colloids). Notably, the peak positions of the experimental data correspond well with the calculated extinction spectra (theory/experiment Au: 523 nm/524 nm, AgAu: 476 nm/471 nm, Ag: 408 nm/407 nm), further supporting that small, homogeneous alloy NPs with a composition similar to the bulk alloy foil formed. In a previous work on laser‐generated alloy particles, the formation of a quasi‐solid solution with a ≈10% composition gradient only on the outermost atom layers was demonstrated,^[^ ^21^, ^26^ ^]^ which contrasts the chemical synthesis route where the formation of gold‐enriched cores is typical.^[^ ^27^ ^]^ Furthermore, target composition and particle composition were proven to be identical on a single particle level.^[^ ^21^ ^]^ Please note that AgNP were solely synthesized and analyzed as controls for optical characterization of AgAu NPs and not exposed to the bacteria due to their known adverse effects on the bacterial membrane, which would impair systematic uptake studies.

### 2.2 Incubation of the Nano‐Bioconjugates with *S. aureus*

**Figure 2:**
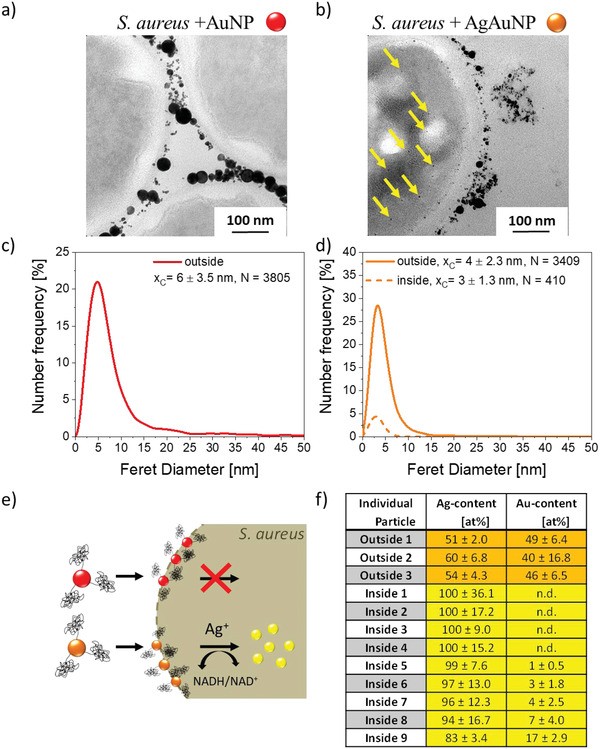
Behavior of colloidal Au and AgAu nanoparticles after incubation with *S. aureus*. Electron micrographs showing the accumulation of Au NPs at the bacterial cell surface but: a) no internalized NPs and NPs found inside only in the case of: b) AgAu alloy NPs. Nanoparticle size analysis of extra‐ and intracellularly located particles (Number‐weighted particle size distributions were derived from N individual particles located inside or outside the bacterial cells, *x*_c_ values represent number‐mean values, and errors were calculated from the variance of the log‐normal fit functions) (c,d). Pathway of Au NPs and AgAu alloys schematically shown in (e). Elemental composition of intra‐ and extracellularly located particles after incubating *S. aureus* with AgAu NPs (f).

**Figure 3:**
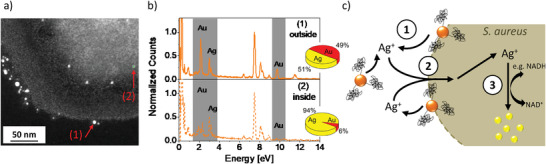
HAADF‐STEM micrograph of an *S. aureus* cell incubated with AgAu NPs. Visualization of particles close to the outer bacterial cell wall and particles located within the cell. Note that brighter contrast corresponds to heavier material (a). Elemental composition of selected extra‐(1) and intracellular (2) particles (b) extracted from EDS measurements. Proposed three‐staged REPER‐mechanism: (1) Extracellular dissolution and RElease of Ag^+^ cations from AgAu alloy NPs, (2) PEnetration of Ag^+^ cations into cells, and (3) intracellular Reduction to pure Ag NPs (c, step 3).

After physicochemical characterization, AgAu and Au colloidal nanoparticle‐aptamer conjugates were incubated with *S. aureus*, a potentially pathogenic gram‐positive bacterial strain (**Figure** **2**), to investigate the cellular uptake behavior. While *S. aureus* usually acts as a commensal bacterium, the medical relevance is particularly due to the emergence of biofilm‐forming *S. aureus* and antibiotic‐resistant strains such as *MRSA* (methicillin‐resistant *S. aureus*).^[^ ^28^ ^]^ Particle size distributions inside and outside the cells were analyzed via TEM. Further representative TEM images of *S. aureus* cells incubated with AgAu and Au NPs can be found in Figures S1 and S2 (Supporting Information), respectively. Please note that a small total number of larger particles (20–30 nm) is detectable in the TEM images (compare Figures 1 and 2). They, however, do not show in the number‐weighted particle size distributions as their total number is too low. As next to their low abundance in number their specific surface area is also smaller than that of the particles <10 nm, we did not consider these minority fraction particles in consecutive mechanistic considerations. The higher abundance of these larger NPs in the pure gold control samples in the presence of bacteria (Figure 2A) may also be the result of an aggregation process in the presence of the bacteria or a size‐selective accumulation of larger particles on the bacterial walls, an observation which may be interesting to study in future works. Regarding the incubation with Au NP conjugates, electron micrographs showed that particles did not enter the cell but accumulated at the bacterial cell wall (Figure 2a,b), probably attributed to the presence of the targeting aptamers. No change in the average particle size could be observed between as‐prepared, aptamer‐conjugated NPs and those accumulated around the cells (Figures 2c and 1e). In contrast with pure Au, the utilization of AgAu alloy particle conjugates revealed the presence of metallic NPs inside the *S. aureus* cell (**Figure** **3**). Moreover, internalized particles featured a smaller size and narrower size distribution compared with those outside the bacterial cells (Figure 2d). To get a comprehensive picture of the intrabacterial particle fate, energy‐dispersive X‐ray spectroscopy in TEM (TEM‐EDX) was used to examine if the chemical composition of internalized and noninternalized particles deviates. Most interestingly, the material composition of particles inside the cells was completely different from the composition of particles outside the cells (Figure 2f). While external particles showed AgAu compositions close to the original 50/50 value, internalized particles consisted primarily of silver (>90% Ag).

Since Au NP and AgAu alloy NP conjugates of comparable sizes were employed, the differences in NP uptake cannot be explained by a size effect. This is backed by our observation that some particles with similar sizes were found outside (*d* = 3.5 nm) and inside the cell (*d* = 2.6 nm), which only deviated in composition (Figure 3a). Therefore, the material composition seems to dictate cellular uptake (Figure 2e).

### 2.3 Proposed Uptake Mechanism

Based on previously performed detailed investigations on AgAu NP derived from LAL synthesis, we can confirm that alloy nanoparticles are quantitatively formed from alloy targets, while the composition of particle and target are identical.^[^ ^21^ ^]^ Hence, we can exclude that pure silver nanoparticles were present in the sample before they were exposed to the bacteria. The most probable mechanism to explain the presence of silver‐rich internalized particles in the case of aptamer‐coated AgAu alloy NPs is based on the presence of high local concentrations of Ag^+^ cations, which are dissolved from the alloy particles in the cell's vicinity. Subsequently, these Ag^+^ cations probably diffused into the cells and were intracellularly reduced to silver nanoparticles. The postulated REPER mechanism consisting of three steps: (i) RElease of Ag^+^ cations in the bacteria's vicinity, (ii) Ag^+^ cation PEnetration through the membrane, and (iii) internal Reduction to silver nanoparticles is illustrated in Figure 3c.

To further verify and test the hypothesis of an ion‐mediated REPER mechanism, several control experiments were conducted. Initially, we performed silver cation dissolution studies during the incubation of AgAu NPs in a bacteria‐free cell medium quantifying the amount of released Ag^+^ via electrothermal atomic absorption spectroscopy (ET‐AAS). During 96 h, 0.05% of the initially present silver dissolved from AgAu alloy NPs (Figure S4, Supporting Information). This roughly corresponds to the amount of silver detected in the bacterial cells (9.63 × 10^5^ silver cations per bacterial cell); details on this calculation can be found in ESI. Therefore, we conclude that the presence of dissolved Ag^+^ cations in relevant amounts is highly likely within our *S. aureus* system.

To further confirm this hypothesis of a silver cation‐mediated uptake mechanism, we incubated the bacteria with silver salts at concentrations corresponding to the same amount of silver present in the used AgAu NPs. This incubation demonstrated the presence of small intracellular nanoparticles (Figure S5, Supporting Information). Based on these findings, we conclude that an ion‐mediated mechanism is highly likely to be involved. In another series of control experiments, we aimed to elucidate the role of the surface aptamers. To that, we initially incubated the bacteria with unconjugated AgAu NPs. These experiments showed fewer particles accumulated at the bacterial cell membrane, and no intracellular particles could be found (Figure S3, Supporting Information). We also performed control experiments with AgAu‐aptamer conjugates containing the miniStrep aptamer with no known affinity for the bacterial membrane. In this experiment, we found an even lower affinity of the AgAu NPs for the surface of the bacteria than in the unconjugated controls and primarily agglomerated NPs detached from the cell membrane were found (Figure S6, Supporting Information). This phenomenon is probably attributed to the anionic character of the aptamer, which, in case specific binding is omitted, leads to repulsive electrostatic interactions with the cell membrane.^[^ ^29^ ^]^ Another point of interest is to which extent the aptamer itself would affect the transport of the formed silver ions into the bacteria. To this end, we incubated Ag^+^ salt solutions with the free thiolated aptamer. In this case, TEM analysis did not show any internalized small NPs but larger NPs situated outside of the bacteria (Figure S7, Supporting Information). This phenomenon can be attributed to two overlapping effects. First, free aptamers, due to their anionic character, can easily complex and bind the free silver cations, a phenomenon, which might interfere with the binding of the aptamers to the bacterial membrane. Second, the free thiol groups in the aptamers could be responsible for the reduction of the Ag^+^ cations forming larger AgNPs. Finally, we aimed to elucidate whether nanoclusters with diameters <3 nm are formed during the incubation of AgAu alloy NPs with *S. aureus*. Thereto, we centrifuged the samples after incubation of the bacteria with the AgAu‐aptamer conjugates and analyzed the supernatants via analytical ultracentrifugation, a technique well‐suitable for the detection of nanoclusters.^[^ ^30^ ^]^ The resulting particle size distributions clearly show particle size fractions with several local maxima between 0.5 and 2 nm, which is clear evidence for nanocluster formation (Figure S8, Supporting Information). As nanoclusters are present after incubation of the AgAu‐conjugates, their involvement in the formation of the intrabacterial AgNPs cannot be excluded. It is conceivable that Ag nanoclusters also form extracellularly in the presence of aptamer ligands with complexing and reductive properties, penetrate the bacterial membrane, and serve as seeds for the growth of the intracellular AgNPs.

The findings from the previously presented control experiments confirm the hypothesis of an ion‐mediated uptake mechanism. However, they also indicate that accumulation of the corresponding ion‐releasing NPs in the vicinity of the cells by targeting ligands and the corresponding high local concentration of silver cations are necessary prerequisites to observe this phenomenon. Furthermore, the presence of nanoclusters in the supernatants indicates that cluster‐sized intermediates may be involved, a finding that warrants further research. In this context, it should be noted that we observed the REPER mechanism in the presence of small AgAuNPs with diameters <5 nm. As the mechanism requires a high local concentration of released silver ions in the vicinity and silver ion release is favored by a high specific surface area, it is probably only possible to observe this phenomenon in larger NPs when they are applied at a substantially elevated mass concentration. In addition to the experimental evidence presented above, the dissolution of AgAu alloy nanoparticles has been intensively studied by others. In this regard, selective dissolution of Ag^+^ cations may either de‐ or increase in the AgAu alloys compared with pure Ag NPs. A passivating effect was described by Besner et al. and Pratsinis et al.^[^ ^9^, ^31^ ^]^ This effect was attributed to altered electron‐transfer properties in the nanoalloy (i.e., a shift of the chemical potential), where gold atoms may form internal electron traps, inhibiting the oxidation of silver and the release of Ag^+^ cations.^[^ ^11^, ^28^ ^]^ On the other hand, enhancement of silver cation release and increased antimicrobial activity was described for bimetallic AgPt and AgAu/polymer nanocomposites compared with the pure Ag/polymer composite.^[^ ^32^ ^]^ The authors attribute the effect to the galvanic coupling of Ag to the more noble metal Au when the two are in electrical contact, which is in line with results from Link et al. that revealed the dissolution kinetics of single AgAu nanoparticles.^[^ ^12^ ^]^

Subsequently, membrane penetration of Ag^+^ via ion channels might occur, which has been reported before.^[^ ^33^ ^]^ The third step involving the formation of NPs inside cells is backed by numerous reports on the biogenic NP synthesis by reduction of metal salts in plants, algae, fungi, bacteria, and viruses (e.g., reviewed by Thakkar et al.).^[^ ^34^ ^]^ In general, the reduction or precipitation of toxic metal ions to insoluble, nontoxic metal clusters can be regarded as a microbial detoxification mechanism. Different mechanisms have been proposed for the intracellular reduction of metal ions involving enzymes, carbohydrates, and biomembranes.^[^ ^35^ ^]^ Small thiols, glutathione, fatty acids, or polyphosphates have been suggested to act as templating agents in *E. coli* for the bacterial biosynthesis of cadmium sulfide nanocrystals from cadmium chloride and sodium sulfide salts.^[^ ^36^ ^]^ The involvement of nitrate reductase in the formation of Ag NPs from silver cations was demonstrated in vitro and was suggested to be involved in microbially mediated Ag NP synthesis in the soil bacterium *Bacillus licheniformis* and the marine fungus *Penicillium fellutanum*.^[^ ^37^ ^]^ Since nitrate reductase is also part of the respiratory system of *S. aureus*, this could explain the intracellular reduction of silver cations previously dissolved from AgAu alloy NPs and the formation of small Ag NPs inside the cells observed in our experiments.^[^ ^38^ ^]^ The experimental results allowed us to draw a clear mechanistic picture of silver‐based nanoparticle uptake in bacteria. In brief, the REPER mechanism involves three steps (Figure 3c): (i) RElease of Ag^+^ cations in the bacteria's vicinity, (ii) Ag^+^ cation PEnetration through the membrane, and (iii) internal Reduction to silver nanoparticles. Please note that this mechanism may have been responsible for nanosilver found inside bacteria during studies of other authors, though our approach based on AgAu NPs could verify it, as we could differentiate intra‐ and extracellular compositions of the NPs.

## 3 Conclusion

In summary, this study investigated the fate of aptamer‐conjugated AgAu alloy NPs after incubation with the pathogenic bacterial strain *S. aureus*. Interestingly, pure silver NPs not present in the initial sample were found inside the bacteria. The selective and local dissolution of silver cations from AgAu NPs in the vicinity of the bacteria, focused by targeting ligands, and the subsequent cellular uptake of these ions, followed by their intracellular reduction were suggested to account for the presence of small, silver‐rich particles within bacterial cells. Due to the absence of endosomal uptake mechanisms in bacteria, nanoparticle internalization is generally difficult to achieve. With this model system of partly dissolving (AgAu) and nondissolving (Au) colloids as controls, a mechanistic understanding of the effects of colloidal nanoparticles on bacteria could be gained, showing that the selective transport of silver cations into the cells is probably involved, though the presence of nanocluster intermediates with diameters <2 nm may be an indicator of a more complex formation mechanism, which warrants further examinations. Similar ion‐mediated mechanisms could also account for previously observed uptake events from pure silver nanoparticles in bacteria; however, in these cases, particles inside and outside the bacteria had identical compositions, and cellular uptake of particles and silver cations could not be differentiated. In this context, AgAu alloy NPs, which are overall more biocompatible than their silver analog, conjugated with functional molecules like aptamers, could serve as vessels for targeted and specific transport of antibacterial agents like silver cations to the bacteria to combat severe infections locally and selectively.

## 4 Experimental Section

### Laser Synthesis of Colloidal Nanoparticles and Particle Characterization

Colloidal nanoparticles were produced via pulsed laser ablation in liquids.^[^ ^22^ ^]^ A picosecond laser system with a wavelength of 1064 nm, pulse duration of 10 ps, a repetition rate of 100 kHz, and pulse energy of 80 µJ was employed (Atlantic laser, Ekspla). The bulk metal foil (silver, gold, or silver–gold alloy, Research Institute for Precious Metals and Metals Chemistry, Germany) and 30 mL medium (aqueous solution containing 2.5 g L^−1^ BSA) were placed in a self‐designed PTFE batch chamber.^[^ ^39^ ^]^ The medium inside the chamber was constantly stirred, while the laser beam was horizontally focused onto the target and was moved in a spiral pattern via a scanner system (scan speed: 6 m^−1^ s, outer spiral diameter: 6 mm, ScanCube10 scanner, and LaserDesk software, ScanLab) for 20 min.

The optical properties of the colloids were determined by UV/Vis extinction spectroscopy (Thermo Scientific Evolution 201). A measurement range from λ = 300 to 800 nm was chosen, and samples were analyzed in a cuvette with a 10‐mm path length and 1.5 mL volume. Transmission electron microscopy of as‐synthesized nanoparticles was performed using a transmission electron microscope (Morgagni, FEI, Eindhoven, Netherlands) equipped with a digital camera (Olympus Soft Imaging Systems, Münster, Germany) at an accelerating voltage of 80 kV. The results were shown based on logNormal fits of the number‐weighted size distributions. Mean diameters corresponded to the *x*_c_ values of the logNormal distribution ± standard deviations. Average particle diameters were found to be 2.8, 3.0, and 2.3 nm for Ag NPs, AgAu NPs, and Au NPs after laser ablation, respectively. In addition, particle diameters for the incubation of AgAu NPs with *S. aureus* are 3.5 nm (outside) and 2.6 nm (inside bacterial cells).

### Nanoparticle Conjugation with Aptamer Ligands

Thiolated aptamers (Eurofins Genomics, Germany, Sequence: Thiol‐GCAATGGTACGGTACTTCCTCCCACGATCTCATTAGTCTGTGGATAAGCGTGGGACGTCTATGACAAAAGTGCACGCTACTTTGCTAA) designed to target *S. aureus* were suspended in TE buffer (0.01 mol L^−1^ tris(hydroxymethyl) aminomethane, TRIS, Merck, and 0.001 mol L^−1^ Ethylenediaminetetraacetic acid, EDTA, Sigma Aldrich) according to the manufacturer's protocol.^[^ ^23^ ^]^ The miniStrep aptamer (TCTGTGAGACGACGCACCGGTCGCAGGTTTTGTCTCACAG‐T10‐(CH2)3‐S‐S (CH_2_)_6_OH) was used for the generation of nontargeting conjugates as controls. Nanocolloids (19 nM) were suspended by ultrasonication for 15 min and mixed with the suspended aptamers (20 µM) at a volume ratio of 1:1, resulting in aptamer‐to‐NP ratios of ≈950:1. Here, a large surplus of deployed aptamers was chosen to promote targeting of the bacterium. The vial was shaken (400 rpm, 20 °C) for 48 h to provide the conjugation of colloids and aptamers. Please note that conjugation was carried out at a high surplus of the aptamer ligand, which would go along with the saturation of the surface coverage and is expected to be in the range of 140 pmol cm^−2^ as deduced in the previous work.^[^ ^40^ ^]^ Concerning functionality (affinity for the bacterial surface), the authors assumed that a high surface coverage on the NP surface coincided with a maximum functionality for ligand‐free AgAu alloy NP conjugates.^[^ ^41^ ^]^ The vial was subsequently centrifuged at 125 000 *g* for 20 min. The supernatant was carefully removed to avoid disturbance of the particle‐aptamer sediment and then centrifuged at 125 000 *g* for 20 min. After removing the second supernatant, both sediments were mixed in 1000 µL TE buffer.

### Cultivation of *S. aureus* and Preparation of Bacterial Samples for Electron Microscopy

*S. aureus* (DSM 20 231, German Collection of Microorganisms and Cell Cultures, DSMZ, Braunschweig, Germany) were cultivated in Mueller‐Hinton‐medium (CM0405, Oxoid Limited, Hampshire, UK) for 8 h, reaching a phase of exponential growth. After dilution of the medium (OD: 0.1), nanoparticles in suspension were added to the cell medium to a final concentration of c(NP) = 50 µg mL^−1^ and thoroughly mixed. After further incubation for 1 h, the suspension was centrifuged for 2 min (13 000 rpm), and the supernatant was discarded. After further centrifugation with the same parameters, the cell pellet was mixed with low melting point agarose (1%) and centrifuged for 3 min (13 000 rpm). The bacteria pellet was washed three times with 200 mM Hepes (4‐(2‐hydroxyethyl)−1‐piperazine ethane sulfonic acid, pH 7.35, GIBCO, Thermo Fisher Scientific Inc., Waltham, USA) and then fixed in Hepes buffer containing 4% Paraformaldehyde and 0.1% Glutaraldehyde. Following dehydration via an increasing acetone series, samples were embedded in Epon (Serva, Heidelberg, Germany) and contrasted with 1% osmium tetroxide (EMS, Hatfield, PA, USA) and 4% uranyl acetate (Serva, Heidelberg, Germany). Subsequently, the fixed samples were cut into 50 nm thick layers using an ultramicrotome to prepare them for TEM characterization.

### Electron Microscopy of Bacterial Samples

Conventional transmission electron microscopy was performed on 50 nm sections using an electron microscope (Morgagni, FEI, Eindhoven, Netherlands) equipped with a digital camera (Olympus Soft Imaging Systems, Münster, Germany) to obtain images of representative fields of view. As described above, the same TEM specimens were studied using an electron probe aberration‐corrected microscope (FEI Titan G2 80–200) operated at 80 kV and equipped with bright‐field, high‐angle annular dark‐field (HAADF) detectors and in‐column EDX detectors.

### Analysis of ion Release from Ag NPs and AgAu NPs in Biological Medium

Conjugates were prepared as described before with the following modifications: 316 pmol number concentration of NPs (mass‐concentrations: Ag NP 215 µg mL^−1^, AgAu NP 300 µg mL^−1^) were conjugated with 18 nmol aptamers, resulting in aptamer‐to‐NP ratios of 57:1. Conjugates were washed twice, and the pellets were resuspended in TSB medium (30 g Trypton Soy bouillon, Thermo Scientific; 3 g yeast extract, Carl Roth; in 1 L distilled water) in the final NP concentrations of 50 µg mL^−1^ Ag NP and AgAu NP were established by diluting with TSB buffer. Volumes of 5 mL conjugates were filled into dialysis tubes (Float‐A.LyzerG2, Spectrum Laboratories, Inc., 8–10 kDa), and the tubes were immersed into 40 mL TSB medium for ion release experiments. Please note that the aptamer‐to‐NP ratio was lowered in these experiments to reduce the amount of free and highly charged thiolated aptamers, which could complex silver ions and might impair their passage through the dialysis membrane. Ten samples of 0.5 mL were taken within a period of 96 h. The silver content was determined by electrothermal atomic absorption spectroscopy (ET‐AAS). The Ag content of TSB media samples using a 1/10 dilution with 1% nitric acid in deionized water (Millipore‐MilliQ‐Academic) was measured in triplicates. ET‐AAS was performed with a Perkin Elmer AAnalyst‐600 instrument, equipped with a Zeeman effect background correction system. A total of 20 µL of the diluted samples was transferred by Perkin Elmer AS 70 autosampler into a pyrolytic graphite furnace tube with a L'vov platform. ET‐AAS temperature program for Ag was described in detail by Zimmermann et al.^[^ ^42^ ^]^ The Ag standard of 1 g L^−1^ used for calibration was obtained from Bernd Kraft GmbH, Duisburg, Germany.

### Incubation of *S. aureus* with Silver Ions and Analysis of Ion Reduction

*S. aureus* was cultivated in TSB medium overnight, centrifuged for 15 min (4000 *g*), and suspended in 10 mM TRIS buffer (pH 8, OD: 2). The bacteria were then incubated for 1 h with either 78.56 mg L^−1^ silver acetate (AgC_2_H_3_O_2_, Sigma‐Aldrich, St. Louis, USA) or 77.2 mg L^−1^ silver nitrate (AgNO_3_, Sigma‐Aldrich) at 37 °C while being shaken with 200 rpm. Subsequently, 15 mL of 8% formaldehyde and 5% glutaraldehyde in phosphate‐buffered saline (PBS, Sigma‐Aldrich) were added, and the samples were further incubated for 30 min at room temperature. The samples were then centrifuged for 5 min (4000 *g*). The resulting pellets were suspended in 1 mL of 4% formaldehyde and 2.5% glutaraldehyde in PBS and incubated for 90 min at room temperature. The samples were centrifuged for 5 min (4000 *g*), and the fixed bacteria were washed three times for 5 min with PBS. Finally, the pellets were suspended in 1% formaldehyde in PBS for TEM analysis.

### Characterization of Supernatants by Analytical Ultracentrifugation (AUC)

*S. aureus* was incubated with AgAu‐aptamer conjugates and supernatants were separated from cells by centrifugation as specified in above section. Supernatants were analyzed using analytical ultracentrifugation (AUC) (Beckmann Coulter, optima XLI) which gave access to the hydrodynamic particle diameter, with high specificity for nanoclusters with hydrodynamic diameters <3 nm. The authors measured in the sedimentation‐velocity mode under centrifugal field conditions of 11 290*g, 20 °C. Sedimentation coefficients of 0–1127 were used during the measurement and a density of 14.9 *g* * cm^−3^ corresponding to AuAg was assumed.

### Statistical Analysis

TEM images were analyzed based on an individual number of N individual particles (indicated in the corresponding figures). The diameters of these particles were determined using the image analysis software ImageJ. Number‐weighted particle size distributions were generated and fitted with a log‐normal function. Number mean values were derived from this fit function and errors were calculated from the variance of the corresponding fit functions. Ion release experiments were done as triplicates and errors were derived from standard deviations.

## Conflict of Interest

The authors declare no conflict of interest.

## Supporting information

Supporting Information

## Data Availability

The data that support the findings of this study are available from the corresponding author upon reasonable request.
