# Supplementary material for: The Origin of the Intracellular Silver in Bacteria: A Comprehensive Study using Targeting Gold–Silver Alloy Nanoparticles
Source: Adv Healthc Mater. 2023 Sep 17;12(30):2302084. doi: 10.1002/adhm.202302084 (PMC11469222; doi:10.1002/adhm.202302084)
Supplement: Supplementary file 1 — Supporting Information [file ADHM-12-2302084-s001.pdf]

ADVANCED HEALTHCARE MATERIALS

Supporting Information

for *Adv. Healthcare Mater.*, DOI 10.1002/adhm.202302084

The Origin of the Intracellular Silver in Bacteria: A Comprehensive Study using Targeting Gold–Silver Alloy Nanoparticles

*Carmen Streich, Frederic Stein, Jurij Jakobi, Alexandra Ingendoh-Tsakmakidis, Nils Heine, Christoph Rehbock, Andreas Winkel, Sebastian Grade, Mark Kühnel, Vadim Migunov, András Kovács, Thomas Knura, Meike Stiesch, Bernd Sures and Stephan Barcikowski**

Supporting Information

Origin of the intracellular silver: A comprehensive study using targeting AgAu nanoparticles

*Carmen Streich, Frederic Stein, Jurij Jakobi, Alexandra Ingendoh-Tsakmakidis, Nils Heine, Christoph Rehbock, Sebastian Grade, Andreas Winkel, Mark Kühnel, Vadim Migunov, András Kovács, Thomas Knura, Meike Stiesch, Bernd Sures, Stephan Barcikowski***

1. Supplementary results

1.1. Supplementary electron micrographs of the incubation of *S. aureus* with nanoparticles

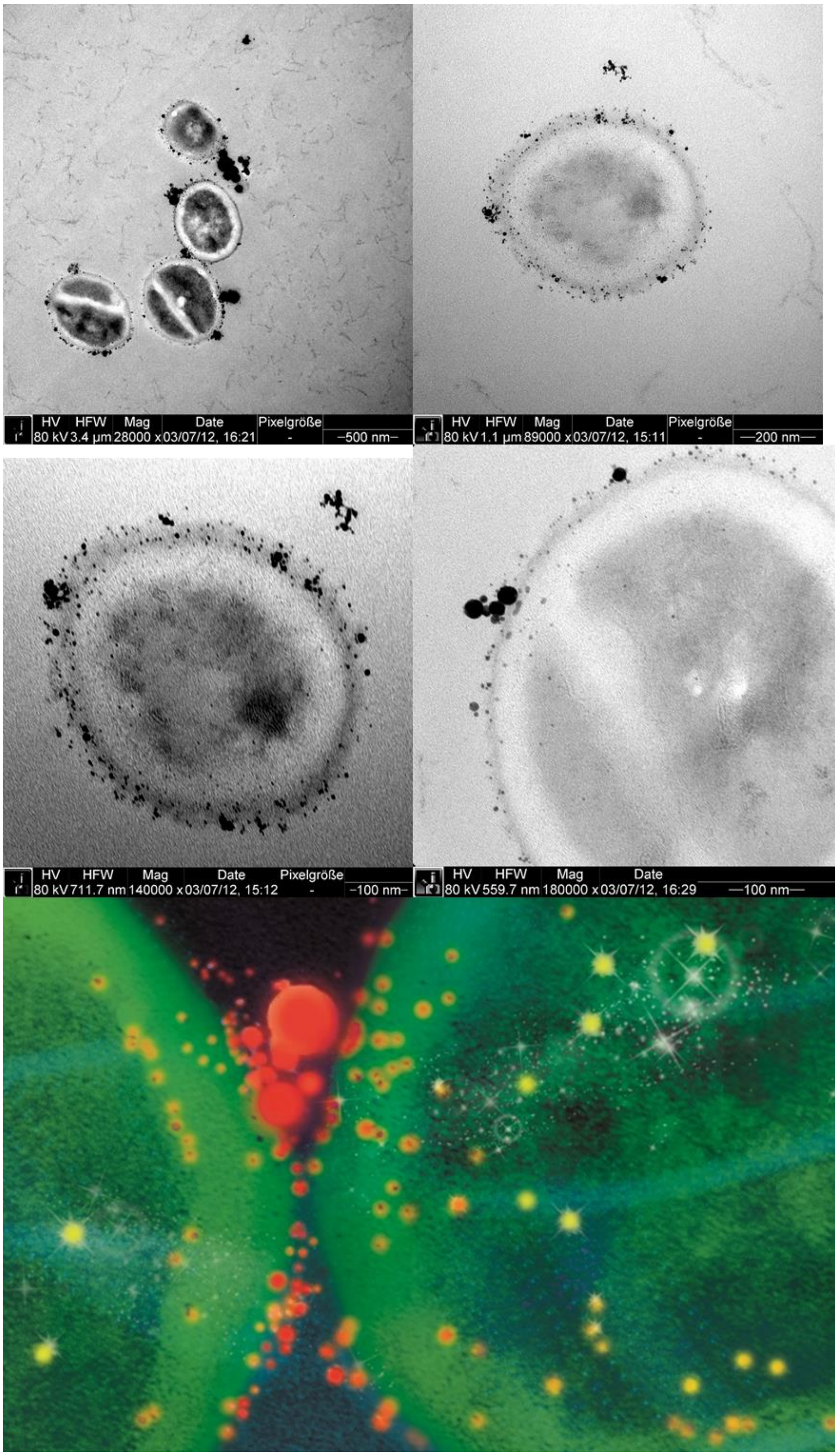

Figure S 1: Top: Representative TEM images at different magnifications showing S. aureus incubated with aptamer-coated AgAu NPs. Black dots indicate regions with high contrast showing metal nanoparticles differentiated from the background of the bacterial cells. Black dots inside the bacteria represent successful penetration. Bottom: Graphical artwork showing a false-color electron micrograph of S. aureus with AgAu NPs (in red) and internalized Ag NPs (in yellow) implementing data from EDX single particle analysis into the image, demonstrating the REPER-mechanism.

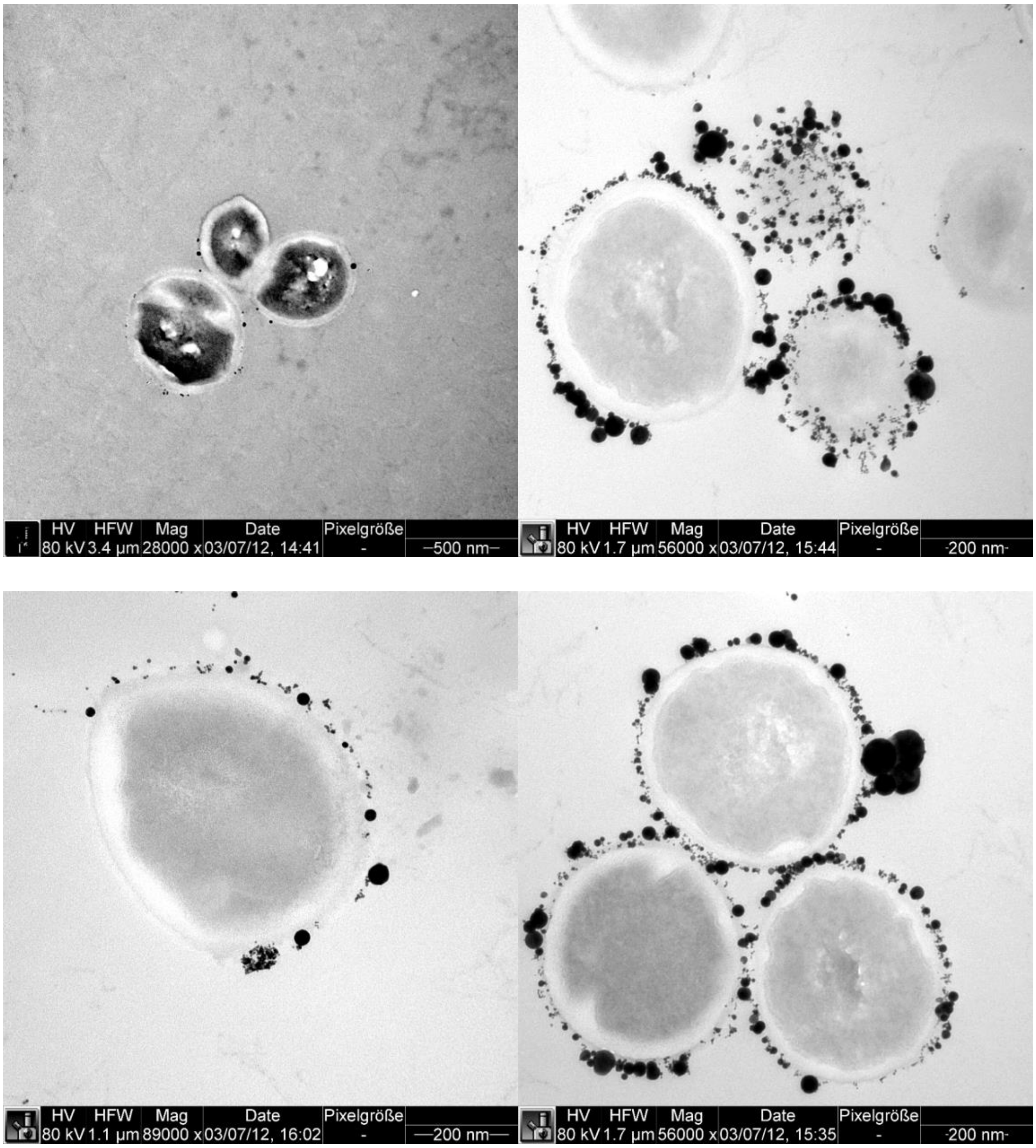

Figure S 2: Representative TEM images at different magnifications showing S. aureus incubated with aptamer-coated Au NPs. Black dots indicate regions with high contrast showing metal nanoparticles differentiated from the background of the bacterial cells.

1.2. Influence of aptamers on nanoparticle uptake

Specific aptamers were coupled to the particles via a thiol bond to enhance interactions with the bacterial membrane. The interactions of thiol groups with gold and silver surfaces and nanoparticles are known to differ.[1, 2] Hence, different aptamer surface coverages may arise for different particle types, such as AgAu and Au NPs. This may lead to differences in the nanoparticles' affinities for the bacterial cell wall. However, these differences are unlikely to account for the element-selective cellular uptake.

The following control experiments were conducted with aptamer-free nanoparticles to clarify if the aptamer is required for nanoparticle transport to the bacteria, adsorption at the cell wall, and uptake into the cells. For this purpose, aptamer-free AgAu NPs were incubated with *S. aureus*.

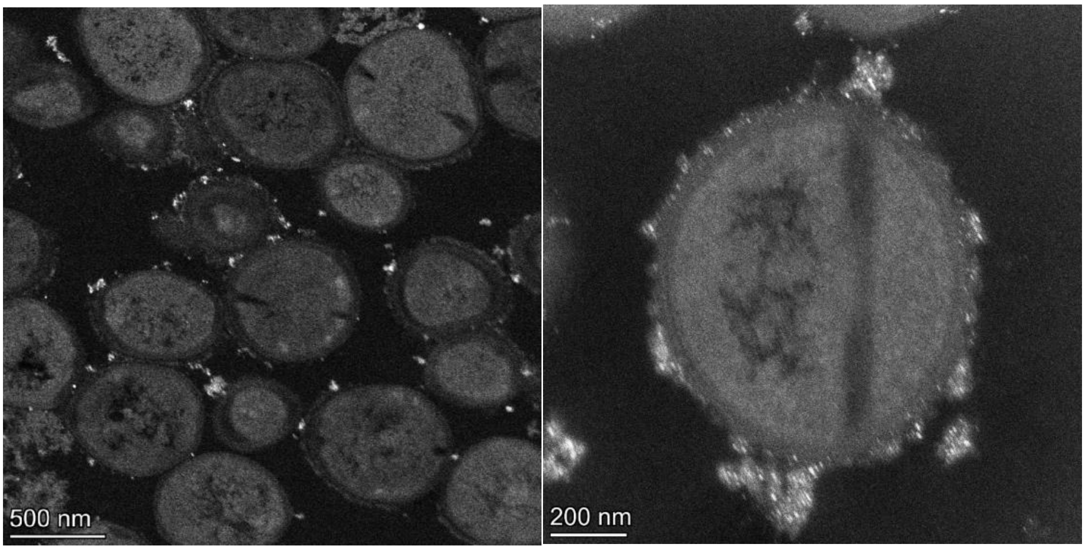

Figure S 3: HAADF-STEM electron micrographs showing S. aureus incubated with aptamer-free AgAu NPs

The TEM micrographs in **Figure S 3** show that incubation of ligand-free AgAu NPs with *S. aureus* results in less NP accumulation on the bacterium's cell wall. Correspondingly no intracellular particles were found when aptamer-free AgAu NPs were used. Since the number of particles in the vicinity of the bacterium is lower, the amount of released silver cations taken up by the cell is insufficient to reduce them to silver nanoparticles (see the mechanistic picture in Figure 3c in the main manuscript).

1.3. Analysis of ion release from Ag NPs and AgAu NPs in biological medium

The dissolution of silver cations from alloy nanoparticles in a biological medium was examined using AgAu alloy nanoparticles coated with aptamer ligands. To spatially separate nanoparticles from ions, colloids were placed into dialysis tubes (V = 5 mL), and the silver cation concentration was analyzed in the medium surrounding the dialysis tubes (V = 40 mL), while aptamer-conjugated Ag NPs were used as a reference.

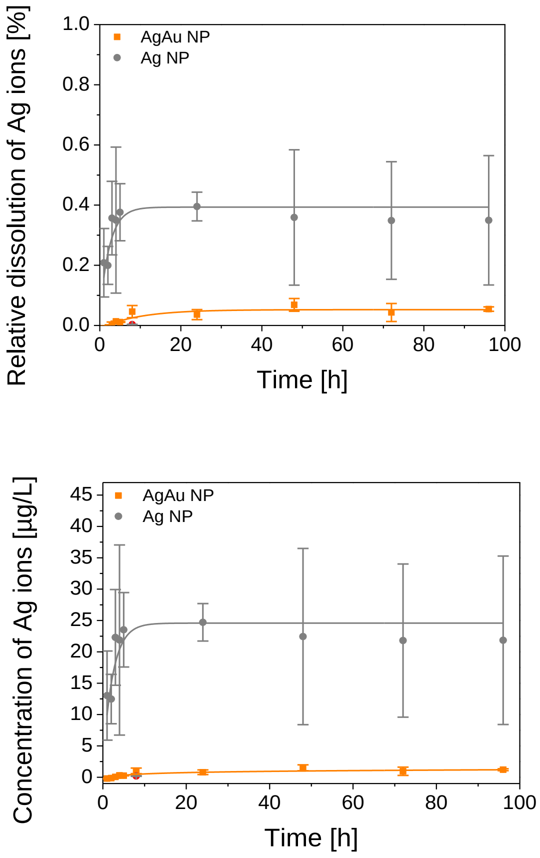

Figure S 4: Dissolution of silver cations from AgAu alloy NPs and pure Ag NPs during the incubation in cell culture medium (without bacteria). The silver cation concentration detected in the cell medium surrounding the dialysis tubes is shown as a relative concentration related to the initially applied silver amount (left) and as an absolute concentration (right). Error bars represent standard deviations, derived from triplicates with different particle samples.

Figure S 4 shows an increase in silver cation concentration over time during the incubation of Ag and AgAu colloids in a cell culture medium. A constant silver concentration of 22.6 ± 1.1 µg/L is reached in the sample containing Ag NPs after 3 h, which corresponds to a dissolution of 0.362 ± 0.017 % of the initially applied silver concentration. In contrast, the samples containing AgAu NPs show a constant silver concentration of 1.1 ± 0.3 µg/L after 8 h, which corresponds to a dissolution of 0.050 ± 0.012 % of the applied silver.

As reported in previous studies, alloying silver with gold alters the oxidation behavior of the less noble metal, which may explain the overall lower silver cation concentration in AgAu alloy NP samples.[3,4] Interestingly, the absolute silver concentration released from the samples containing AgAu alloy NPs in this model experiment corresponds well with the amount of silver,

which is estimated to be present inside the bacterial cells after the cultivation experiment. This silver amount was theoretically calculated as follows:

An average diameter of 1 µm, spherical shape, and a volume of 0.52 μm^3 was assumed for the bacterial cell. Regarding the TEM images of bacterial cells taken from bacterias´ cross-sections, in which the nanoparticles were observed, we estimate the volume of the bacterium to be 0.0045 μm^3 (50 nm (thickness) x 300 nm x 300 nm). Hence, the resulting number of sections per bacterial cell is 116.4. If one detects 10 particles per image section on average, this corresponds to a total number of 1164 NPs per bacterium.

For one spherical Ag NP with the diameter of 3 nm, a volume of 14.1 nm^3, a density of 10.5 g/cm^2, a mass of 1.48 x 10^{-13} µg, and a molecular mass of 108 g/mol, the number of silver atoms was calculated to be 828 per particle.

In the cultivation experiment, the bacterial concentration was OD = 0.1, corresponding to approximately 8 x 10^7 cells/ml. Therefore, the theoretically required silver concentration is 9.63 x 10^5 atoms per bacterium and 7.71 x 10^{13} per ml culture medium. Assuming that 100 % of the silver cations are reduced, this would correspond to a silver concentration of 13.8 µg/L.

During the release experiment with AgAu alloy particles for the incubation in pure cell medium (without bacteria), a silver concentration of 1.1 ± 0.3 µg/L was determined. Considering the dilution with cell medium by a factor of 8, the corrected silver concentration is 8.8 µg/L.

As shown in the calculations, the determination of the silver concentration released from the samples containing AgAu alloy NPs after incubation in cell medium (8.8 µg/L) corresponds well with the amount of silver, which is estimated to be present inside the bacterial cells after the cultivation experiment from TEM images (13.8 µg/L), though admittedly some rough assumptions were used during this calculation.

1.4. Incubation of *S. aureus* with silver cations

Since the aptamer may have an impact on the formation of the intracellular Ag NPs and to verify the ion-driven REPER mechanism, silver nitrate was incubated with *S. aureus* with the

respective same amount of silver present in total in the used AgAu NP, which amounts to a silver cation concentration of 47 µg/ml (**Figure S 5**).

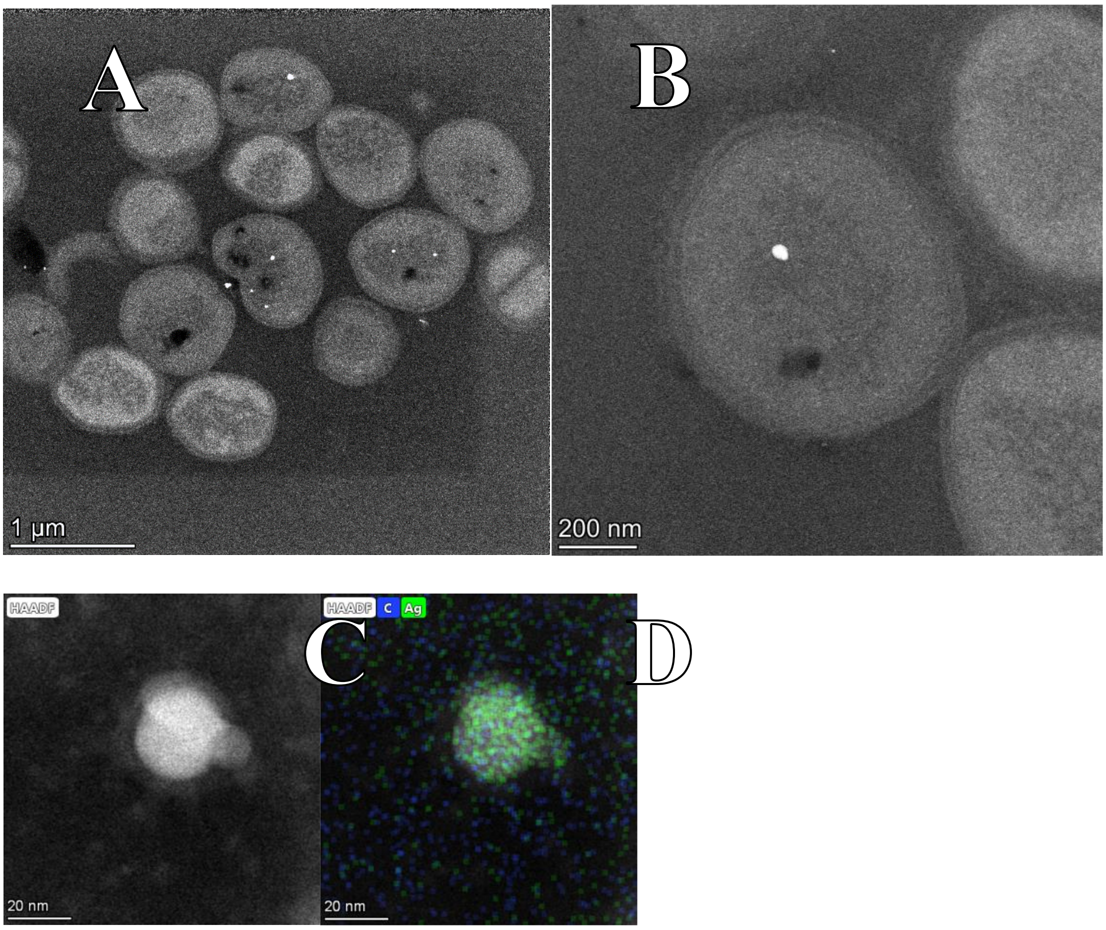

Figure S 5: A, B) HAADF-STEM electron micrographs showing S. aureus incubated with silver nitrate at the respective same silver concentration of the AgAu NPs with silver NPs formed inside the bacteria. C, D) show single-particle EDX mapping of the bright spot in B, confirming the presence of silver.

Ag NPs can also be found within and around bacteria when silver salt instead of AgAu NPs is used, which is a strong indicator of reduction processes occurring in the presence of *S. aureus*. In this case, the Ag^+ cations from the silver salt are entirely available to interact with the bacteria in contrast to the quasi-bound silver in the metallic state of nanoparticles. This leads to higher toxicity and disintegration of bacteria and subsequently to the release of Ag NPs in the environment after they form inside the bacteria. EDX-mapping analysis (see Figure S 5 D) confirmed the presence of pure silver NPs. In context with the previous finding that only a small number of aptamer-free AgAu NPs accumulated at the membrane of *S. aureus* and that no

intracellular particles were found, this confirms the hypothesis that a certain number of silver cations are required concentrated in the bacteria's vicinity to form intrabacterial NPs.

1.5. Incubation of *S. aureus* with non-targeting AgAu-aptamer conjugates

To which extent the specificity of the aptamer sequence affects the interaction between AgAu-conjugate and the bacterial membrane was checked by incubating *S.aureus* with AgAu-aptamer conjugates containing a non-targeting miniStrep aptamer while aptamer/nanoparticle ratio was kept at 950:1.

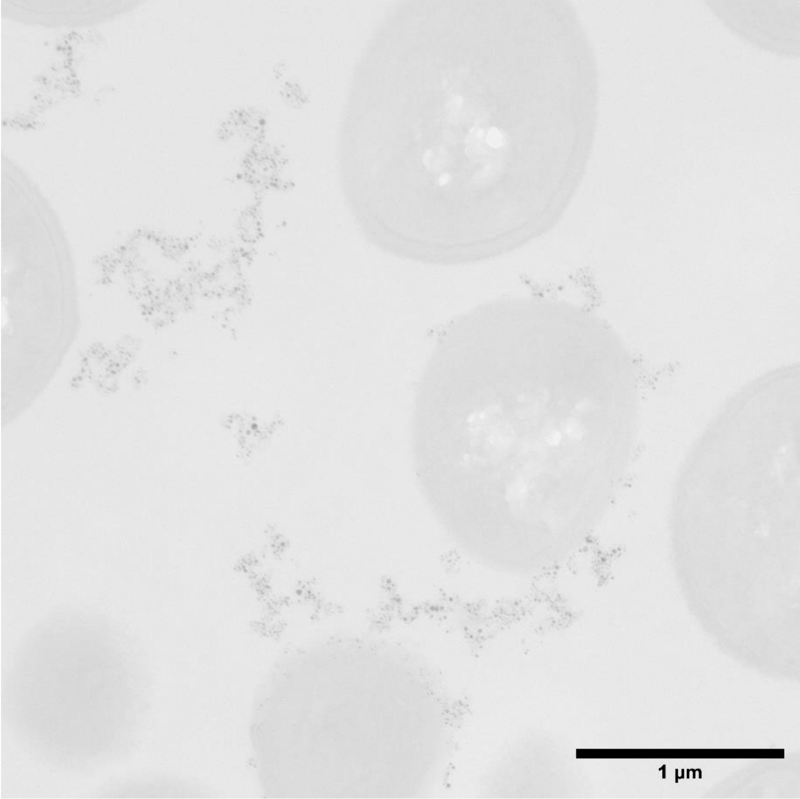

Figure S 6: Representative TEM image showing S. aureus cells incubated with non-targeting AgAu-aptamer-conjugates.

As seen in the TEM image, agglomerates of AgAu NPs can be found in the sample, while attachment to the bacterial membrane is far less pronounced than in all other controls. This indicates that the nature of the aptamer sequence has a strong influence on the binding behavior. In the case of a non-binding aptamer, the anionic character of the aptamer is dominant, which leads to repulsive interactions with the cell membrane omitting attachment and penetration.

1.6. Incubation of *S. aureus* with free aptamers and silver ions

In this experimental series, we aimed to study interactions between the free aptamer and the free Ag^+ cations by exposing *S.aureus* bacteria to a mixture of $AgNO_3$ and the free thiolated aptamer.

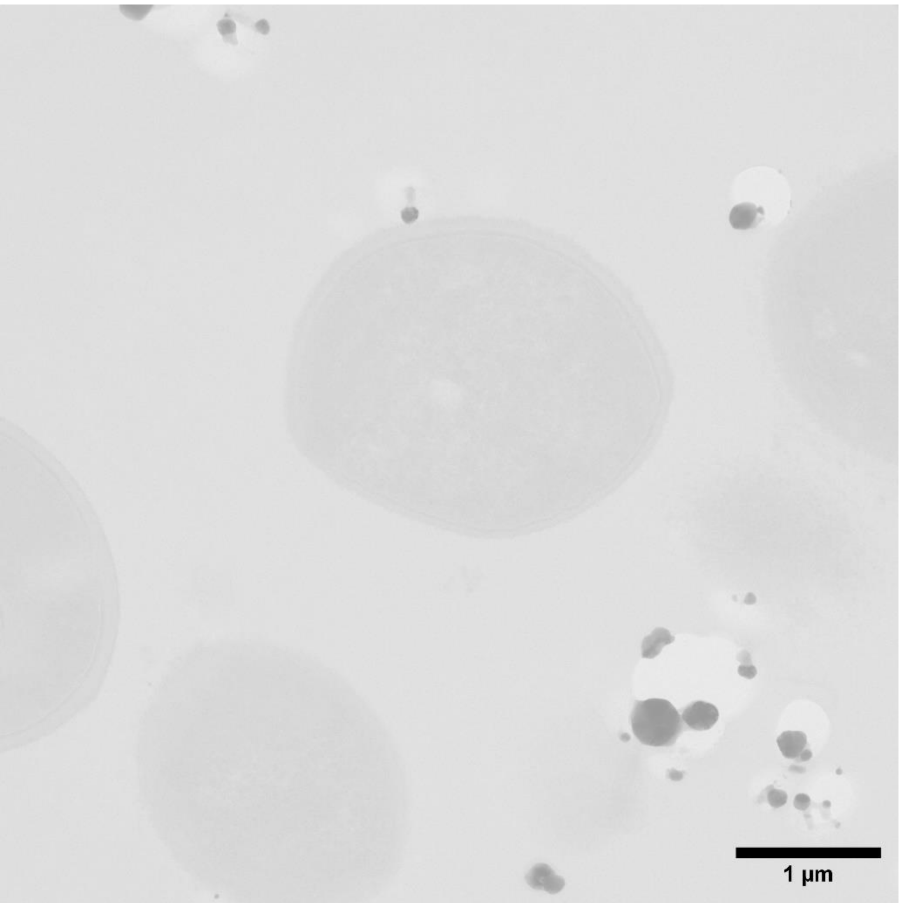

Figure S 7: Representative TEM image showing S. aureus cells incubated with a mixture of $AgNO_3$ as well as a free thiolated aptamer

The resulting TEM images show the formation of larger extracellular NPs. This finding is probably attributed to electrostatic interactions and complexing of the Ag^+ by the negatively charged phosphate backbone of the aptamer, which inhibits strong interactions with the bacteria. The reduction of the silver ions and the consecutive formation of AgNPs is most likely caused by the reductive character of the free thiol group on the aptamer ligands.

1.7. Formation of nanoclusters during incubation of AgAu-aptamer conjugates with *S.aureus*

To deepen our understanding of the formation mechanism of intrabacterial AgNPs we analyzed the supernatants of bacterial samples for the presence of nanoclusters using analytical

ultracentrifugation (AUC). A representative size distribution from AUC in the size regime < 4 nm is shown in Figure S8. The findings verify the presence of particle size fractions with diameters of 0.5 -2 nm.

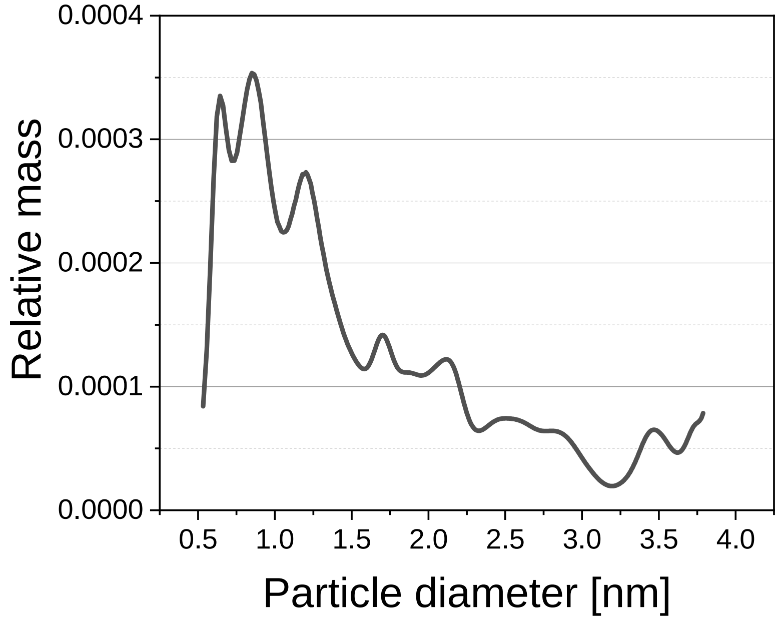

Figure S 8: Volume-weighted particle size distributions from analytical ultracentrifugation (AUC) obtained from supernatants of bacterial samples incubated with AgAu-aptamer conjugates after cells and larger particles were separated by centrifugation.

2. References

[1] N. Maiti, R. Chadha, A. Das, S. Kapoor, RSC Adv. 2016, 6, 62529.

[2] H. Sellers, A. Ulman, Y. Shnidman, J. E. Eilers, J. Am. Chem. Soc. 1993, 115, 9389.

[3] S. Besner, M. Meunier, J. Phys. Chem. C. 2010, 114, 10403.

[4] A. Al-Zubeidi, F. Stein, C. Flatebo, C. Rehbock, S. A. Hosseini Jebeli, C. F. Landes, S. Barcikowski, S. Link, ACS nano. 2021.
